# A qualitative exploration of patient safety in a hospital setting in Spain: Policy and practice recommendations on patients' and companions' participation

**DOI:** 10.1111/hex.13758

**Published:** 2023-04-14

**Authors:** Daniel G. Abiétar, Laia Domingo, Laura Medina‐Perucha, Nuria Saavedra, Anna Berenguera, Laia Lacueva, Marta Hurtado, Xavier Castells, María Sala

**Affiliations:** ^1^ Servicio de Epidemiología y Evaluación Hospital del Mar Barcelona Spain; ^2^ Facultad de Ciencias de la Salud y de la Vida Universidad Pompeu Fabra Barcelona Spain; ^3^ Red de Investigación en Cronicidad Atención Primaria y Promoción de la Salud (RICAPPS) Madrid Spain; ^4^ Fundació Institut Universitari per a la recerca a l'Atenció Primària de Salut Jordi Gol i Gurina (IDIAPJGol) Barcelona Spain; ^5^ Universitat Autònoma de Barcelona Barcelona Spain; ^6^ Unidad de enfermería de Cirugía Ortopédica y Traumatología Hospital del Mar Barcelona Spain; ^7^ Servicio de Metodología y Calidad en Cuidados Enfermeros Hospital del Mar Barcelona Spain; ^8^ Servicio de Atención a la Ciudadanía Hospital del Mar Barcelona Spain

**Keywords:** companions, hospital setting, patient participation, patient safety

## Abstract

**Introduction:**

Patients' and companions' participation in healthcare could help prevent adverse events, which are a significant cause of disease and disability. Before designing interventions to increase participation, it is first necessary to identify attitudes to patient safety. This study aimed to explore patients' and companions' perceptions, attitudes and experiences of patient safety, taking into account contextual factors, such as cultural background, which are not usually captured in the literature.

**Methods:**

We conducted a qualitative study with a theoretical sampling of 13 inpatients and 3 companions in a university hospital in Barcelona, Spain. Information was obtained from individual and triangular interviews. A descriptive thematic content analysis was conducted by four analysts and a consensus was reached within the research team on the key categories that were identified. We also conducted a card‐sorting exercise.

**Results:**

All informants emphasized the role of good communication with health professionals, a calm environment and the need for patient education. Discursive positions differed by cultural background. Informants from a Pakistani–Bangladeshi background emphasized language barriers, while those from European and Latin‐American backgrounds stressed health professionals' lack of time and the need for more interdisciplinary teamwork. The card‐sorting exercise identified several opportunities to enhance participation: checking patient identification and medication dispensation, and maintaining personal and environmental hygiene.

**Conclusion:**

This exploration of informants' discourse on patient safety identified a wide variety of categories not usually considered from institutional perspectives. The findings of this study could enrich interventions in areas with diverse cultural backgrounds, as well as current frameworks based exclusively on institutional perspectives.

**Patient or Public Contribution:**

The results of the study were communicated to patients and accompanying persons via telephone or email. Similarly, a focus group was held with a patient forum to comment on the results. In the design of subsequent interventions to improve patient safety at the hospital, the proposals of patients and companions for their participation will be included together with healthcare professionals' opinions.

## 1 INTRODUCTION

According to the World Health Organization, adverse events due to unsafe care are one of the 10 leading causes of death and disability worldwide.^1^ In high‐income countries, it is estimated that 1 in 10 patients experience an adverse event while receiving hospital care,^2^ of which almost 50% are preventable.^3^ This is also true of Spain.^4^ In this regard, inequities in the safety of care and a higher risk of adverse events have been found in ethnic minorities.^5^ Recommendations for hospital quality improvement, and especially for the prevention of adverse events, usually originate from and are directed towards healthcare teams.^6^, ^7^ However, it is becoming increasingly important to involve patients directly in interventions to enhance patient safety, which could reduce the burden of harm.^6^, ^8^

Although data on the effectiveness of patient participation in patient safety can vary depending on the specific aims pursued,^9^ there is evidence that adverse events can be reduced by involving patients and their companions in medication monitoring,^10^, ^11^, ^12^ and by preventing pressure ulcers, patient falls and surgical infections.^6^, ^13^, ^14^ In contrast, studies assessing patient involvement in hand hygiene promotion among physicians show they are particularly reluctant to speak out when they observe poor hygiene practices.^7^

Patient participation in safety during admission is a key element of patient empowerment and a hospital culture that promotes a new, more proactive patient role.^15^ Both could be achieved by simply enquiring about patients' experiences during hospitalization.^9^ However, before increasing patient participation, there is a need to identify patients' characteristics, such as cultural background, socioeconomic position, gender and age,^15^ as they may influence attitudes towards participation. Indeed, specific mechanisms are required to ensure participation among certain groups, such as ethnic minorities.^5^ To our knowledge, no other studies have investigated how patient safety is perceived, from different cultural perspectives, and how patients can participate in designing patient participatory processes in a hospital setting in southern Europe. The possibility of codesign requires, among other things, exploring patient safety from the patients' perspective. The aim of this study was to explore patients' and their companions' perceptions of patient safety in a hospital setting, and their recommendations for patient involvement aimed at improving patient safety. The results of this study could be useful to design interventions to engage patients in safety during direct care in other hospitals in the publicly funded health system in Spain.

## 2 METHODS

This qualitative study is part of a larger project conducted in our hospital aiming to design a set of interventions to improve patient safety in hospital care by involving patients and their companions. The project is called ‘Improving patient safety through the active involvement of patients and companions’.

This study was conducted from design to analysis from the perspective of critical theory and equity. This was essential because the research team was aware that the area served by the hospital has inequalities influencing participation and is more deprived than other areas of the city.^16^

### 2.1 Study setting and design

The Hospital del Mar is one of four public university hospitals in the city of Barcelona and attends medium‐ and high‐complexity diseases in a catchment area of more than 300,000 inhabitants. The hospital has more than 400 conventional beds, and 12 operating rooms, and annually assesses more than 95,000 patients in the Emergency Department.

This exploratory and interpretative qualitative study used a naturalist‐comprehensive paradigm. The perspective adopted was socioconstructionist: forms of participation are proposed from particular historical, social and individual contexts, and the aim of researchers is to critically interpret the social positions underlying these proposals.^17^ Data collection techniques consisted of individual and triangular semistructured interviews with patients and their companions. Triangular interviews (also known as triangular groups) are composed of a maximum of three people. They are intended to delve deeper into some topics, because, in a larger group, due to group pressure, the discourse would be less rich. Our triangular groups consisted of interviewing patients and companions together.^17^ In accordance with the project's objective of proposing actions to improve patient safety in the hospital, the degree of data interpretation was high.

An external company gave support in the transcription and coding stages. The external company is a strategic consultancy specializing both in innovation and qualitative studies. They have led trend studies, ethnographic studies, digital ethnography and interviews with experts. A coding procedure was agreed upon before the start of the study. Coding quality and control mechanisms were also established, and the coding was finally reviewed by the research team. A process of sharing the research objectives and the methodological approach was necessary since the company was not involved in the process of designing the study protocol. Patients and companions were told they could ask to stop the interview if they became tired or uncomfortable. Likewise, if interviewers perceived any difficulty, they spontaneously asked if the informants wanted to stop the interview.

### 2.2 Study population

To guarantee appropriate health status for participation, eligible participants were hospitalized patients who were close to discharge and who belonged to European, Pakistani–Indian–Bangladeshi or Latin‐American communities and were older than 18 years, irrespective of the reason for admission. Those selected were also able to understand and provide consent to participate. Patients' companions who were present at the time of the interview were approached after the patient met the criteria and were invited to participate and to provide consent.

### 2.3 Recruitment and sampling

First, participants were identified a priori through hospital records in November 2021, on the basis of general characteristics such as age, sex and place of birth. Among those patients who were expected to meet the heterogeneity criteria, potential participants were approached by two investigators (D. G. A. and N. S.) and by a cultural interpreter (when necessary), face‐to‐face and a short survey was conducted to characterize their profile with the heterogeneity criteria described in the paragraph below. A convenience sample of participants was recruited in‐house (D. G. A.) after the short survey was conducted. Sample diversity and the equity perspective were ensured by using the following heterogeneity criteria: age (18–34, 35–64 and >65 years); a sense of belonging to a cultural community (European, Latin American, Indian–Pakistani–Bangladeshi and Maghrebi); educational attainment (none, primary education, secondary education, vocational training and higher education); sex assigned at birth (male, female or intersex) and gender identity (feminine, masculine or nonbinary*: nonbinary people are those who do not identify with the male–female binomial). People from non‐Western cultural communities were specifically included to avoid underrepresentation due to their lack of access to effective participation. The cultural communities selected are the four most frequent in the health areas served by the hospital. Only one potential participant refused to participate in the study because she felt dizzy due to her health status.

### 2.4 Data collection

Individual interviews were conducted by the main researcher (D. G. A.) and another researcher from the external company in the patient's hospital room. We interviewed 16 participants (13 hospitalized patients and 3 companions who were with patients when they were first approached). Therefore, 10 were individual interviews and 3 were triangular. A cultural mediator participated in interviews with a cultural and/or language barrier. The interviews were conducted in Spanish (*N* = 10), Urdu (*N* = 2) and English (*N* = 1) and lasted between 20 and 50 min.

**Figure 1:**
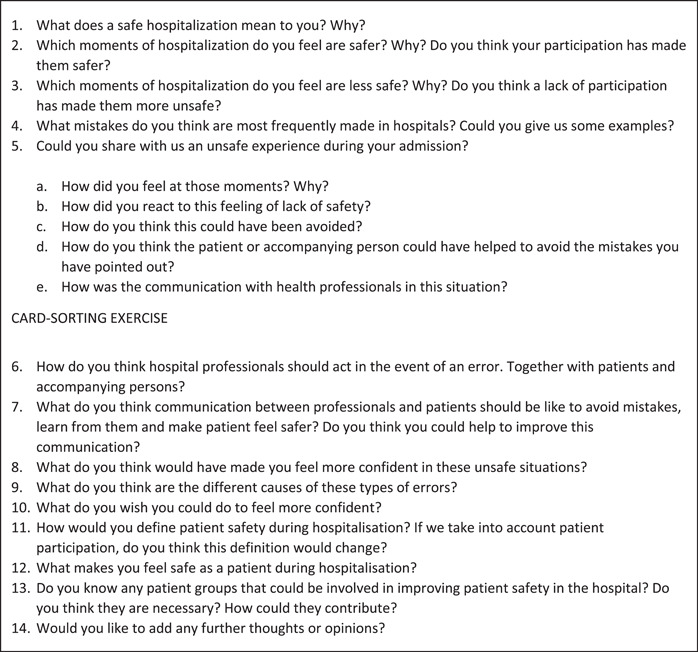
Topic guide used in the interviews (16 participants) taking place in Hospital del Mar (Barcelona) between November and January 2021–2022.

**Figure 2:**
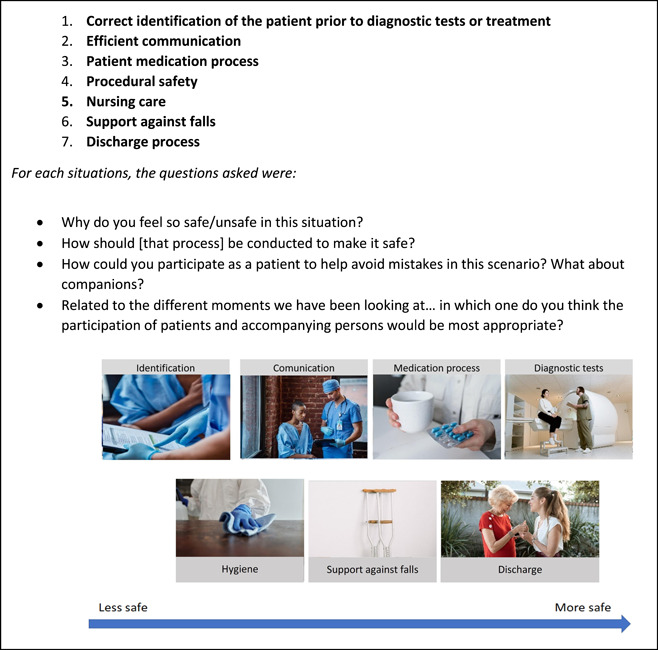
Card‐sorting exercise. ‘Here are some situations in which a patient like you may feel unsafe. Rank them according to your perception from least safe to most safe and explain why’.

Study participants were asked to describe the following in‐depth: (a) the concept of patient safety, (b) their safe and unsafe experiences during hospitalization and the possible causes and (c) their proposals to improve patient participation in patient safety. We used a semistructured topic guide (Figure 1) to conduct the interviews, based on findings from a nonsystematic review of qualitative and quantitative studies examining patients' perspectives and experiences of patient safety in hospitals, created by four members of our team. Because most previous studies have been presented within frameworks consistently based on professional perspectives, in this study, we deliberately chose not to use a specific theoretical framework, as a means to explore how patients and their companions conceptualized patient safety in their own words. During the interviews, and after question 5, a card‐sorting exercise was used to facilitate visualization of safety‐related scenarios (Figure 2) and to try to overcome language and health literacy barriers. The informants were asked to rank the images from those representing the least safe situation to that representing the safest situation and to explain their reasons as a way to help them to think about these situations. All the information collected with this exercise was included in the textual corpus for the descriptive thematic content analysis.

All interviews took place in December 2021 and January 2022 and were audio recorded with the participants' permission. Data were collected until saturation criteria were reached. We considered the discourse ‘saturated’ when discursive positions were clearly defined and no new elements were identified for any of them.

### 2.5 Data analysis

The audio recordings were transcribed verbatim by an external professional. Two sessions were held between the external professional and two researchers (D. G. A. and M. S.) to check the coding process and carry out the handover for the rest of the analysis. Then, a descriptive thematic analysis was carried out by three researchers (D. G. A., L. D. and L. M.‐P.).^18^, ^19^ They began by reading the transcripts to identify the range of data in the data set, and then first independently, and then jointly, performed the following steps: (i) identification of the relevant subjects and texts; (ii) fragmentation of the text; (iii) text codification with emerging codes; (iv) creation of subcategories and categories; (v) analysis of each category and (vi) interpretation of the emerging findings. The results were subsequently discussed among the research team (D. G. A., L. D., M. S. and L. M.‐P.) until reaching a consensus on the key categories.

Because the discourse of the selected cultural communities had not been previously explored in our setting, we did not decide on a specific conceptual framework beforehand for the data analysis.

## 3 FINDINGS

Of the 16 participants, 11 (69%) were aged between 35 and 64 (range: 21–80) years. Seven informants (44%) had elementary education or no schooling. Community belonging was heterogeneous (five European, six South Asian and five South American). Nine informants (66%) were men and 7 (44%) were women. None of the participants identified as nonbinary or trans (gender identity) or reported being intersex (sex at birth). Patients were from different hospital services: general surgery, gastroenterology, neurology, neurosurgery, urology, oncology, traumatology and internal medicine. Patient characteristics are shown in Table 1.

**Table 1:** Patient characteristics.

Participant^a^	Age category	Reasons for admission	Cultural community belonging	Educational level	Sex assigned at birth	Gender identity
(1) Patient A	>65	Colostomy	Spanish	Primary school	Woman	Feminine
(2) Patient B	35–64	Epilepsy	Latin‐American (Argentina)	Primary school	Man	Masculine
(3) Patient C	35–64	Hernia	Spanish	Vocational training	Woman	Feminine
(4) Companion A (patient D's companion)	35–64	Patient D had multiple pathologies (COVID‐19 and cancer)	Pakistani	None	Man	Masculine
(5) Patient E	>65	Spondylodiscitis	Spanish	Higher education	Man	Masculine
(6) Patient F	35–64	Septic arthritis	Pakistani	Don't know	Man	Masculine
(7) Patient G	35–64	Lithiasis	Latin‐American (Brazil)	Secondary school	Woman	Feminine
(8) Patient H	18–34	Appendicitis	Pakistani	Higher education	Man	Masculine
(9) Patient I	18–34	Femur fracture	Pakistani	Secondary school	Woman	Feminine
(10) Patient J	35–64	Anaemia	Latin‐American (Ecuador)	Primary school	Man	Masculine
(11) Patient K	35–64	Pneumonia	Bangladeshi	Primary school	Man	Masculine
(12) Patient L	35–64	Subarachnoid haemorrhage	Latin‐American (Brazil)	Secondary school	Woman	Feminine
(13) Patient M	35–64	Hernia	Spanish	Primary studies	Woman	Feminine
(14) Patient N	35–64	Postgastrectomy haematoma	Latin‐American (Argentina‐Paraguay)	Secondary studies	Man	Masculine
(15) Companion B (patient M's companion)	35–64	–	Spanish	Primary studies	Woman	Feminine
(16) Companion C (patient I's companion)	18–34	–	Pakistani	Higher education	Woman	Feminine

^a^ Names have been anonymized fictional to protect participant's identities.

**Figure 3:**
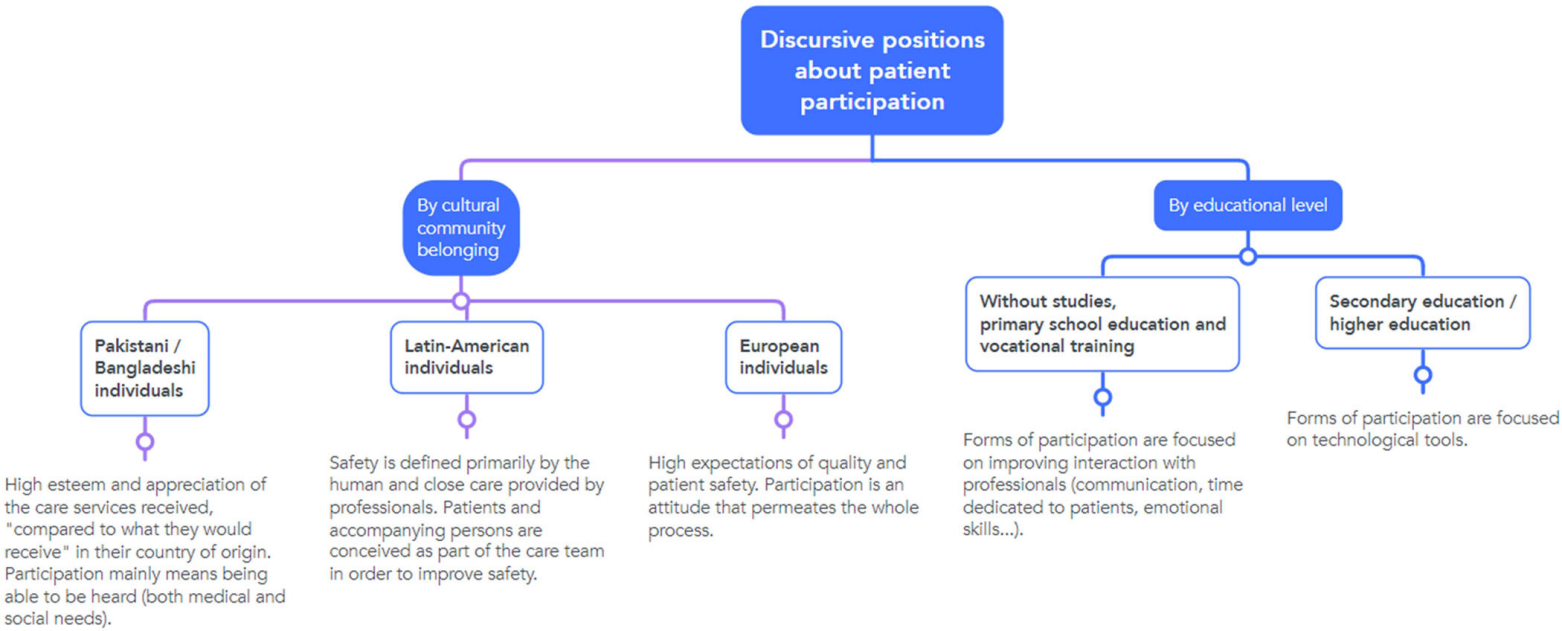
Discursive positions on patient participation, depending on two different axes (sense of community belonging and educational level).

We identified three major categories: (i) conceptualization of patient safety and key moments of safety and insecurity; (ii) factors related to the institution, health professionals and informants influencing informants' participation and (iii) informants' proposals for their participation in patient safety. The latter two points were dominated by two axes: cultural community and educational level, which determined patient‐professional interaction, and the degree of acculturation (Figure 3). The three major categories are presented below, together with participant quotes.

### 3.1 Conceptualization of patient safety and its key moments

For all informants—independently of cultural background, age, sex assigned at birth and educational level—their conceptualization of patient safety during hospitalization was broader and more ambiguous than the concept commonly used by preventive medicine services (‘absence of errors in healthcare’) and was specifically defined in subjective, relational terms.

#### 3.1.1 Subjective factors

When asked for a definition of patient safety, informants reported, on the one hand, positive experiences of trust and well‐being between patients and health professionals, mostly with nurses and, on the other hand, also negative experiences, such as shame during wound care or a lack of communication by physicians. Therefore, they emphasized the value of the subjective component of safety. In their discourse, they assigned little importance to the usual definition of patient safety, understood as an awareness of errors and experiences of adverse events, because in one patient's words, ‘we trust the professionals, otherwise we wouldn't be here’. In other words, if informants trusted professionals, they trusted them not to make mistakes.

#### 3.1.2 Safety understood as quality

To a certain extent, the informants' conceptualization of safety was similar to the concept of ‘quality’, and they had both positive and negative perceptions during hospitalization. For participants, patient safety was mostly related to two dimensions: professionals' behaviour (e.g., ‘humane manners, professionalism, sincerity and honesty’ and coordination as a team), as well as a comfortable hospital environment (e.g., ‘clean and quiet space’, and ‘being supported’). They reported that both items protected them from errors and made them feel safe. Perceptions were closely linked to the discursive positions shown in Figure 3. As stated by patient B:

Good doctors, good nurses […]. For example, the doctors express themselves very well, the nurses are nice. The beds are good… sometimes they give beds that break your back, and here they don't, for example. And more things, I don't know how to explain it, the socialization between patient and nurse or between patient and doctor and so on

For the informants, calm and cordial relationships among all the hospital staff were important, not simply relationships among the healthcare staff or patient‐professional communication. This idea was expressed by patient L:

In the hospital there are people living together: the cleaning team, the cook, the doctors, the nurses. It is a family and these people are like ants, they walk around and do their work. From the moment they say ‘Good morning′ to me, or the cleaning lady works happily, or the person who brings the food is friendly and happy… I know that everything is going well. From the moment people are bitter, shouting, swearing, the doctor humiliating the staff… it's bad for us [patients]. There are hospitals where that happens, I haven't seen it here, but there are. There are places where you work but you are oppressed. And this influences the patient's treatment. However, when the team works together, the result is felt by the patient

#### 3.1.3 Key moments for safety

Based on the participants' accounts during the card‐sorting exercise, most participants perceived that some phases were safer than others. For all of them, the safest part of the admission was the stay in the hospital ward, and the most unsafe and critical moments were waiting for admission and transfers, especially among those with language barriers and with no formal education, or only primary schooling or vocational training (Figure 3). Several needs were identified during these periods. Some informants mentioned communication barriers during transfers around the hospital—both a lack of signage/signaletics and interpreters for Pakistani/Bangladeshi communities. As stated by patient F's interpreter:

He is happy now, he says that the worst was on Monday in the emergency room, when he was left alone. He says he was in a wheelchair and waiting for a long time, but no one could help

Other informants, like patient C, mentioned the need for more information at discharge:

When I get out of here I want to know what I have to do, what my life is going to be more or less like now, because of course I'm not supposed to be able to make any effort or anything. Maybe they explain it to me and I say ‘ok, yes’, but I don't understand. I need help from someone else

### 3.2 Factors influencing patients' and companions' participation

Informants felt that patient participation was mainly influenced by the following subcategories (i) patient and companion factors (‘knowledge’ or ‘cultural background’); (ii) infrastructures and institutional factors (‘patient spaces beyond rooms’, time for patient feedback face‐to‐face and ‘professional's working conditions’) and iii) professionals' factors (‘communication, emotional management’). Although informants were not specifically asked about factors that might influence patient involvement in safety, these factors spontaneously emerged when proposals were made for involvement, mostly as resistance to the possibilities for patient and companions' participation, rather than as facilitators. Participants often justified their lack of participation by citing structural issues influencing professionals' attitudes towards participation (i.e., lack of staff time to become involved in proposals).

#### 3.2.1 Patient and companion factors

Informants identified several factors that could influence patient participation, most of which were related to patients' attitudes to professionals (seen as empathy, although only some used this word), their knowledge of the institution and the disease (health literacy) and their disease status. Lack of knowledge of the field of patient safety was one of the first barriers mentioned by patients, most of whom felt that they had ‘no legitimacy to say what should be done’. Participation was seen as an ad hoc and management issue, rather than a cooperative and longitudinal one. The issue of health literacy was clearly expressed by patient C:

I've received three interventions, not one. This could have happened to anyone. Could it have been a mistake? Possibly. I can't blame anyone if I don't know [what happened]

Some patients, especially those with no education or with primary education only (but not those with vocational training) and those from Pakistani‐Bangladeshi cultural backgrounds, seemed to consider their participation as passive (‘just to take heed of the recommendations’). This was expressed clearly by patient J:

[He was asked about proposals for participation] It's all good, for my part it's all good. I must listen. If you don't listen…once doesn't usually hurt but…I have to listen to what she says [his nurse]

This view contrasts with participation as active and mediated by technological tools, as mentioned by people with higher educational levels and Spanish backgrounds and exemplified by patient E:

Staff check‐in and check‐out [of the room] could be improved. Here I think it is an improvement that affects people's rights a little bit, but that could be solved with the new software programs

Among patients from non‐European cultural backgrounds, the factors seen as heavily influencing their participation were language barriers (and the presence or absence of a cultural interpreter) and the cultural gap between them and health professionals. This was explained by patient F through his interpreter:

Intr: He told me it's difficult [to participate] because he is a foreigner and they don't know the language [Urdu]. But he says he has been well looked after. He says sometimes there are little things that can happen, because if they are busy and you need them, you must wait a bit, but he says these are little things that don't really bother him. […] He says he's had constipation and he hasn't been able to explain it

Paradoxically, some informants associated this cultural gap with better, rather than worse care, as stated by companion A:

[companion A explained that some exceptions were made in visiting restrictions for Urdu‐speakers patients during the COVID‐19 pandemic due to language barriers with healthcare professionals] Sometimes we [patients and companions] have thought that they treat you better if you are a foreigner. It's not like something is missing, it's not 80 out of 100. Out of 100 I would say it's 100 or more

#### 3.2.2 Institutional and professional factors related to patient participation

In terms of infrastructure, all informants believed that to facilitate patients' participation during admission, hospital spaces should be comfortable (even with background music in the case of a Cuban woman), clean, and safe… with the aim of making them feel ‘at home’ and promote the relationship between patients. In this sense, patient G commented:

Ask him [the patient] what he likes to listen to, because I stopped playing my music to play music for my partner and he was very relaxed, much better. The thing is that, in here, with the coexistence in this little piece that you see here […] that is very important, patients should talk [with their companions]. This curtain only closes when the nurse comes in, but once she leaves it is open and here we are a family, which is better than fighting

#### 3.2.3 Institutional factors

The informants' discourse revealed that professionals are embedded in institutional dynamics, which concern human resources management. In the measures proposed, closely related to time as a resource, two further and relatedideas always were identified: Professionals needed more time to improve patient involvement, but the lack of human resources and their difficult working conditions (salary and schedules) made it ‘practically impossible’ to implement these measures, especially among nursing and auxiliary teams, as health professionals ‘couldn't be more dedicated to their work’. In this regard, the ‘system’ (ambiguity) was criticized for not giving health professionals the opportunity to work as well as possible. The idea was illustrated by patient E:

Now, it's a pain in the ass because of the health care cuts. If there are cuts, everything is shaken. In other words, a person working here cannot earn a miserable salary. Some of them. If that doesn't improve, we are taking health care backwards. […] We are human beings and, even if you have a vocation… forgive me because it brings tears to my eyes to talk about this… even though it's a vocational job, if a person who works here doesn't earn a living wage, it affects their work. The essential work of the country has been neglected. […]

#### 3.2.4 Professional factors

Latin‐American patients in particular identified emotional management skills as a crucial professional‐related factor and believed that they determined how professionals requested their participation. Professionals' reactions to patients' and their companions' doubts and questions made a difference in their attitudes towards participation in different situations and to their perceptions of safety. As mentioned by patient G, both patients and their companions find themselves in very difficult emotional situations:

For me it is very important that health professionals have true vocation, so that patients don't feel mistreated. Because when you're on your hospital bed, you are nervous, and it's easy to break into tears. […] Health professionals can never lose their temper. Because I depend on them, I am in their hands

### 3.3 Patients' and companions' proposals for their participation in patient safety

From the descriptive thematic content analysis, various activities and moments were considered appropriate for participation.

#### 3.3.1 Checking correct identification

The informants believed that the identities of both patients and professionals should be checked, whenever the latter establish contact with them or with accompanying persons, as mentioned by patient C:

Now, if they call me Pepita, I'll say ‘excuse me, my name is not Pepita’. But when they call you by your name from the beginning, and they describe what's wrong with you, it's because they know who you are. […] Patients must give clear and correct information about themselves

#### 3.3.2 Ensuring that the medication dispensed is correct

The participants believed that patients or their companions should check that the medication is correct before taking it and should write down its indication and dosage. This was expressed clearly by patient G:

When they are giving the medicine, ask what this pill is for, to be informed. ‘This one you have to take every day, this one every eight hours’

#### 3.3.3 Proactively helping as much as possible with nursing care, whenever possible

As stated by patient N and patient C, respectively:

I know that to lift a patient up, nurses are small, sometimes they cannot. And if you can help with this and push up…

The first thing when you meet the professional at the visit is to create this bond between professional and patient. And ask how things are going and try to be as communicative as possible. I think that builds trust

#### 3.3.4 Always expressing doubts, giving feedback of safety and being empathetic with health professionals, especially when they perceive errors

Some of these ideas were expressed by patient B

The fact that I am always very affectionate. I am always friendly with people, I think that also helps. […] I also hear that the nurses and assistants are caring, and that also encourages the patient, for example, and lifts his or her spirits. […] The patient should be kind to the doctor and the nurse. And try to understand what they are going to do to you, you are here for a reason

#### 3.3.5 Changes to patient evaluation surveys and the informed consent process

Despite not being mentioned in the card‐sorting exercise, most participants believed that both quality/patient safety evaluation surveys and the informed consent process should change to improve participation. The informants suggested that both should be done entirely orally and face‐to‐face so that patients would have a real opportunity to express doubts. They also believed their experience would be substantially improved if they knew the approximate time of the nurse's appointment during the admission process.

#### 3.3.6 The importance of companions as mediators

Informants reported that companions were important to mediate between the patient and professionals when needed by the patient or when misunderstandings occur, but also when patients cannot deal with some responsibilities (hygiene, being communicative…). This was perfectly explained by patient L:

With the family, communication and diagnostic procedures are important. […] I think communication must be the same with them, with sincerity and in a simple way. […] I am good at taking medication. If you tell me that I have to take a medicine at 8 o'clock, I will take it at 8 o'clock. But there are people who don't, who want to take it at 10 o'clock. For example, if a patient doesn't want to take a shower, but has a cut, if he doesn't clean it, he gets infected. There are people who don't have hygienic habits and for their wounds to heal well, they have to keep them clean and dress the wound. The family has to mediate in these cases

#### 3.3.7 The importance of patient groups

From a collective point of view, patient participation was mainly conceived as interaction with other inpatients. As exemplified by patient N, informants suggested the need for physical spaces designed for patients and their companions, so that they could share experiences during admission and create self‐help links.

I think that patient groups are important. For example, when I started this whole process for the operation, there were patients who formed groups and they are always in contact. I think they are good to be in communication, to know how other patients are doing, how their operations went, to keep this link…

To facilitate patient relations, informants believed that appropriate spaces should be created. This was described by patient L:

An open area in the hospital for people to walk around. Because here [in the rooms] it is closed, there is nothing open, very closed. We could have an area with trees, tables, chairs to sit and talk. So that people can have a life closer to the reality that they have at home

Finally, informants were asked to propose specific measures to be considered for our context, to avoid ambiguity over how patients should participate in patient safety and to prioritize areas of action, which is in agreement with other studies.^6^, ^20^ The proposals are summarized and compared with those in similar studies in Table 2. In these proposals, the effect of the card‐sorting exercise should be considered, as the informants' collective beliefs have been fuelled by the images proposed as moments when patient safety is important.

**Table 2:** Most relevant proposals for participation by study informants and equivalent proposals are discussed in the literature reviewed.

Proposals for patient and companions' participation in this study	Equivalent proposals and their rationale in other studies
Checking *correct identification* of patients and professionals, whenever the latter establishes contact with them or with accompanying persons.	− Also mentioned in Vaismoriadi^20^ and Park.^6^
*Ensuring the medication* dispensed is correct before taking it, writing down notes about its indication and *dosage*.	− To engage both patients and companions in treatment surveillance, de Jong^10^. − Examples of medication management have been described by Gabe et al.^21^ and Jordan et al.^22^ − Successful interventions to encourage patient participation in the monitoring and self‐management of medication in hospitals have been described by Hall et al.^23^
*Proactively helping with nursing care* whenever possible.	− This proposal could be considered together with patients’ and companions' roles in preventing pressure ulcers.^4^
*Always expressing doubts and being communicative* with professionals, especially when patients or their companions perceive errors.	− Patient participation relies on patients being encouraged to raise doubts without fear of offending healthcare staff. Agreement on how patients should ask these questions would encourage patient‐provider trust.^24^ − To encourage patient participation, the management system must be supportive and continuously identify and correct all the weaknesses and failures that arise in the system.^25^ Management should also be committed to supporting and empowering patient involvement and challenging power inequities.^24^
*Maintaining personal and environmental hygiene*.	− Most concerns are about environmental factors such as noise at night and poor bathroom facilities.^5^ − This was also considered important for surgical infections.^4^
*The informed consent process should be* communicated entirely *orally and face‐to‐face to allow patients to express doubts*.	− Communication and cooperation between patients and healthcare professionals are important resources for patient participation in quality improvement projects.^7^
*Professionals ask for patients' safety perceptions at discharge to improve the processes*.	− Successful quality improvement interventions can be simple. Direct patient feedback can pinpoint areas of harm not previously noticed and can embolden healthcare professionals' to report harm, leading to changes at the microsystem level.^7^ − For this type of intervention, professionals might need training.^24^ − Active intervention is required, whether at the individual or collective level, to create an environment where patients are listened to, and their views are taken seriously and acted on. Productive communication does not occur by itself.^5^ − Many problems and interventions in healthcare are complicated or complex, but effective safety interventions can also be straightforward. For example, to encourage patients and their families to report harm, the introduction of a simple, real‐time bedside questionnaire enhanced the ward's overall safety culture.^7^
Participation requires resources (especially time) for professionals. Knowing the approximate *time of the nurse's appointment time* during the admission process could help and their companions.	− Healthcare managers are responsible for providing an appropriate and positive environment for nurses to engage patients in patient safety.^24^ − Research has identified multiple barriers that need to be identified and considered by organizations when managing quality improvement efforts. Such barriers include healthcare system financing, competing organizational changes and the work environment, for example, time constraints, staffing, routines, educational skills and the existing attitudes and culture.^7^
The role of *companions'* is conceived as acting as mediators with professionals when misunderstandings occur or when patients must responsibility.	− This role has been previously conceived, especially when a patient's ability to participate is reduced by illness.^24^ − Companions are expected to speak up at a time when patients themselves are too vulnerable or unwell to act as their own advocates.^5^
*Interaction with other inpatients in physical spaces designed for patients and companions*, so that they can *share experiences* during admission and generate *self‐help links*. Patient associations raise doubts about their usefulness and possible hidden interests.	− It is important to generate a participation culture.^13^ − It is also important to consider patient participation from a collective perspective.^5^ Well‐designed collective forums for the patient and public participation can be a motor for change. The power of the collective contrasts with the inequalities between patient‐professional communication at the point of care, when patients are particularly vulnerable. In collective forums, patients could potentially work together to achieve stronger influence than is possible in individual‐level interventions, which are inherently asymmetrical.^5^ − An easy way to improve quality would be to facilitate face‐to‐face meetings, encourage participants to listen to each other and to reflect, and encourage the development of these relationships and cooperation methods.^7^

## 4 DISCUSSION

### 4.1 Summary

In this study, we explored patients' and companions' knowledge, perceptions and attitudes regarding patient safety in a hospital setting, and their recommendations for patient participation aimed at improving patient safety from an equity perspective and different discourse positions. Independently of informants' characteristics, the factors important for patient safety were transparent, easy‐to‐understand and respectful communication by health professionals and a calm environment. Errors did not emerge as relevant. Informants' conceptions of participation differed, mainly according to their cultural background and educational level. When these last two characteristics could hamper communication with professionals, the discourse was limited to communication barriers and their consequences for participation. When there were no communication problems (European and Latin‐American backgrounds, and secondary and university education), informants added that participation was mainly influenced by a lack of clinical knowledge, the feeling that health professionals worked in suboptimal conditions and were overly busy, and their lack of emotional skills.

### 4.2 Comparison with existing literature

To a lesser extent, the perception of patient safety for patients and companions was defined more on the basis of care structures or outcomes of care than on the healthcare process, which is consistent with the existing systematic reviews on quality.^24^ As in other studies, informants were aware of the existence of errors but did not consider them as the main issue in patient safety and, when they did occur, they mostly blamed poor communication before and after the error (as did health professionals).^6^, ^24^ From our study, this patient's logical reasoning could be interpreted as if communication regulates patient safety conceptualization and perceptions, making errors visible and more important for patients when bad communication is happening, probably because trust in health professionals become deteriorated.

In this regard, several factors that encouraged patients and their companions to develop trust in healthcare teams have been reported in other studies.^7^, ^25^ In our consideration, these factors may include the historically passive role of patients in healthcare, their blind trust in healthcare professionals and the perception of comfort and good quality of care compared with less well‐equipped healthcare services in low‐income countries. As it could be interpreted by patient C's second statement, the development of trust can also be influenced by a lack of knowledge about the disease itself and its clinical management.^26^ Regardless of its causes, in the absence of errors perceptible by the informants, trust was a sufficient reason to feel safe. This process could be interpreted as an act of handing over collective responsibility to protect patient safety to the institution and its professionals. Therefore, these elements could be key in promoting a shift to a more proactive attitude of informants to participate.

One of the most notable results of this study is that there was agreement that transfers were key unsafe moments during hospitalization. However, informants expressed different needs according to their profiles. Older people signalled a need to improve the hospital's signalectics to help with admission, visits and discharge, as reported by the systematic review by Sutton et al.,^7^ whereas the Pakistani and Bangladeshi communities perceived language barriers as hampering transfers during admission, although they also stressed the usefulness of the permanent availability of a cultural interpreter. However, in the existing literature,^6^, ^12^, ^15^, ^27^, ^28^ patients' characteristics are usually not reported, hindering identification of the discursive positions behind the expression of this need.

For some patients, particularly those with a university, high school or vocational education, trust in professional teams was independent of whether they engaged in checking and verifying behaviours (e.g., of medication). Underlying this idea, as proposed in other studies with heterogeneous patient profiles, there is a belief that patients should be able to trust they are receiving competent care, as opposed to assuming a leadership role in their safety.^6^, ^20^, ^29^

Much has been written about factors influencing patient participation, but studies performed in other hospital contexts such as Switzerland, the United Kingdom, the United States and Asia, do not describe patient characteristics.^20^ This hampers comparisons, as it does not answer the question of ‘who says what?’. Patients' knowledge of their disease has previously been noted to be an important factor in participation,^6^, ^7^, ^20^, ^26^, ^30^, ^31^ especially in long‐term diseases, and patient education has been proposed as a plausible solution that could even reduce adverse events.^15^ This entails viewing patient participation as a learning process.^20^ Although experiences and beliefs have been proposed as key factors for participation,^20^ our informants assigned them little importance when they asked about them. Moreover, patients' health conditions are also important and must be taken into account to adapt expectations and proposals for participation.^20^, ^26^ In this respect, it is also obvious but important to remember that some patients may not wish to be actively involved for legitimate reasons.^6^, ^20^ Indeed, as described in Section Methods, 2, one woman refused to participate due to dizziness.

Although improving patients' knowledgeand experiences and deconstructing beliefs would improve patients' willingness to participate, it might be insufficient. On the one hand, from an equity perspective, health professionals in our setting probably lack cultural competency, in addition to the existence of language barriers, which may explain the discourse of Pakistani and Bangladeshi informants concerning participation, and echoes the reflections in a systematic review.^26^ Information from different cultural contexts is valuable to achieve patient involvement.^15^, ^20^ However, some of the studies in this area limited participation to persons with no language problems, which is an ethical problem of the first magnitude. On the other hand, the informants themselves identified institutional and professional factors preventing participation: both the lack of specific resources to allow professionals to devote time directly to talking to patients and their high‐stress working environment. Previous studies have reported that the impact of participation could be limited unless steps are taken to ameliorate cultural, structural and organizational barriers^7^, ^20^, ^24^, ^30^ and, as discussed, patients and their companions are able to recognize them. In this regard, in agreement with other studies,^24^ patients (independent of their educational level or gender identity) identified the importance of emotional management and communication skills in both health professionals and patients for patient safety. As reported by studies conducted in Australia,^32^ this might also ameliorate the negative effects of carers' involvement, by assigning a higher value to their contributions. In our study, Latin‐American informants, who placed special emphasis on having warm relationships with health professionals, were especially sensitive to a lack of emotional management and communication skills. Patient empowerment depends on feeling valued, safe and motivated to participate.^20^, ^33^

### 4.3 Strengths and limitations

This study has some strengths and limitations. First, we did not include any inpatients from Maghreb, which is the third largest non‐Spanish cultural community in the hospital healthcare area. However, we were able to include informants from Asia Minor, Latin America and Europe, and we ensured a diversity of profiles with different educational levels, cultures and gender. There was also a lack of younger participants, given that inpatients are usually elderly people. No exclusions were due to language barriers. Broad characterization of the informants allowed for a richer interpretation of the units of analysis, without neglecting the position of the informants, and enhanced the issuing of recommendations for participatory practices in patient safety.^24^ Despite the presence of a cultural mediator, the cultural gap between the research team and participants from Asia Minor, hampered the detection of variations in cultural practices and could have impaired data collection in this group. This could explain why no differences were found between the countries included in this group and also limits the conclusions of the study in this population. In any case, studies of patient participation in patient safety so far have usually neglected to characterize informants' profiles and have not been inclusive.^7^, ^24^ In addition, for the first time in our setting, we included the role of companions in participation, which, based on fieldwork observations, seems to have facilitated participants' expression of their views. Nevertheless, because of COVID‐19 restrictions, visits were strictly limited, which hampered our ability to interview more companions.

Second, we conducted the interviews during hospital admission, which may have limited participants' responses to more positive opinions about professionals and the healthcare process in general. Moreover, as previously mentioned, limits on visits made it difficult to interview more companions. The inpatient setting could also have led to some processes and structures emerging more frequently than outcomes as indicators of patient safety.

Finally, it was difficult to find quiet spaces for the interviews, although, as previously mentioned, informants could stop the interview at any time. However, performing the interview during the admission facilitated the participation of all patient profiles and avoided the loss of information due to memory bias.

## 5 CONCLUSION

The results of this study indicate that identifying patients' and companions' perspectives on patient' safety and their participation is needed before beginning to design an intervention. Examples shared in our paper show the need for a broader conceptualization of patient safety in hospitals, an explicit description of the cultural characteristics of the communities served and a particular focus on communication between patients, companions and professionals. Both patients and companions are able to identify needs particular to their diverse profiles and key aspects for their involvement in hospital safety.

## 6 IMPLICATIONS FOR POLICY AND PRACTICE

Because informants defined patient safety as the subjective perception of comfort with the hospital environment and trust in health professionals, beyond an academic definition of safety, patients' and companions' perceptions could be another essential element of patient safety evaluation through short specific surveys, as they perceive other dimensions sometimes missed by professionals. Both minor adverse events and patients' and companions' safety perceptions are legitimate and interrelated areas of hospital patient safety. To promote participation from an equity perspective, certain mechanisms such as cultural mediators/interpreters or health education might be needed.

## AUTHOR CONTRIBUTIONS

Daniel G. Abiétar led the initial nonsystematic review. Daniel G. Abiétar, María Sala, Laia Domingo and Laura Medina‐Perucha performed the study design. Daniel G. Abiétar, Laura Medina‐Perucha and Anna Berenguera designed the questionnaire. Daniel G. Abiétar and Nuria Saavedra carried out the fieldwork. Daniel G. Abiétar, Laura Medina‐Perucha and Laia Domingo triangulated the results. Daniel G. Abiétar led the preparation of the original draft of this article, and all authors made contributions by reviewing it.

## CONFLICT OF INTEREST STATEMENT

The authors declare no conflict of interest.

## ETHICS STATEMENT

All participants signed an informed consent form. No identifying names or other details that could have compromised anonymity were used. The study was approved by CEICm‐PSMar (2021/10070/I).

## Data Availability

The data that support the findings of this study are available on request from the corresponding author, María Sala. The data are not publicly available due to ethical issues and if they are asked, containing information that could compromise the privacy of research participants would be deleted before.
